# The oncogenic neurotrophin receptor tropomyosin-related kinase variant, TrkAIII

**DOI:** 10.1186/s13046-018-0786-3

**Published:** 2018-06-18

**Authors:** Antonietta Rosella Farina, Lucia Cappabianca, Pierdomenico Ruggeri, Luciana Gneo, Cristina Pellegrini, Maria-Concetta Fargnoli, Andrew Reay Mackay

**Affiliations:** Department of Applied Clinical and Biotechnological Sciences, University of L’Aquila, L’Aquila, Italy

**Keywords:** TrkAIII, Alternative splicing, Neuroblastoma, Oncogenic signaling, Unfolded protein response, Warburg effect, Hallmarks of cancer, Therapeutic approaches

## Abstract

Oncogenes derived from the neurotrophin receptor tropomyosin-related kinase TrkA act as drivers in sub-populations of a wide-range of human cancers. This, combined with a recent report that both adult and childhood cancers driven by novel oncogenic TrkA chimeric-fusions exhibit profound, long-lived therapeutic responses to the Trk inhibitor Larotrectinib, highlights the need to improve clinical detection of TrkA oncogene-driven cancers in order to maximise this novel therapeutic potential. Cancers potentially driven by TrkA oncogenes include a proportion of paediatric neuroblastomas (NBs) that express the alternative *TrkA* splice variant TrkAIII, which exhibits exon 6, 7 and 9 skipping and oncogenic-activity that depends upon deletion of the extracellular D4 Ig-like domain. In contrast to fully spliced TrkA, which exhibits tumour suppressor activity in NB and associates with good prognosis, TrkAIII associates with advanced stage metastatic disease, post therapeutic relapse and worse prognosis, induces malignant transformation of NIH-3T3 cells and exhibits oncogenic activity in NB models. TrkAIII induction in NB cells is stress-regulated by conditions that mimic hypoxia or perturbate the ER with potential to change TrkA tumour-suppressing signals into oncogenic TrkAIII signals within the stressful tumour microenvironment. In contrast to cell surface TrkA, TrkAIII re-localises to intracellular pre-Golgi membranes, centrosomes and mitochondria, within which it exhibits spontaneous ligand-independent activation, triggering a variety of mechanisms that promote tumorigenicity and malignant behaviour, which impact the majority of cancer hallmarks. In this review, we present updates on TrkAIII detection and association with human malignancies, the multiple ways TrkAIII exerts oncogenic activity and potential therapeutic approaches for TrkAIII expressing cancers, with particular reference to NB.

## Background

The neurotrophin receptor tropomyosin-related kinase A (TrkA) regulates responses to the neurotrophins NGF and NT3 in a wide variety of normal tissues and is critical for normal development and function of both nervous and immunological systems [1, 2].

The first human TrkA-derived oncogene, Trk-oncogene, was identified in colon cancer as a novel chimeric fusion between truncated tropomyosin and protein tyrosine kinase sequence [3]. This preceded TrkA proto-oncogene characterisation as an NGF receptor [4] and the identification of numerous TrkA-derived oncogenes in a wide-range of human cancers. TrkA oncogenes are now considered important drivers in subpopulations of a wide variety of human cancers and, therefore, represent important therapeutic targets [3–18]. Recently, adult and childhood cancers driven by novel chimeric TrkA-fusion oncogenes have been reported to exhibit profound and long-lived therapeutic responses to the Trk inhibitor Larotrectinib [18], highlighting a need to improve clinical detection of cancers driven by activated TrkA oncogenes in order to take full advantage of novel Trk-inhibitory therapies [13–18].

Cancers potentially driven by TrkA oncogenes include paediatric neuroblastomas (NBs), a significant proportion of which express the oncogenic alternative *TrkA* splice variant, TrkAIII. TrkAIII exhibits exon 6–7 skipping and is subject to spontaneous ligand-independent intracellular activation, dependent upon deletion of the extracellular receptor D4 Ig-like domain, encoded within exons 6 and 7, which acts as a spontaneous-activation prevention domain [19]. In contrast to fully spliced TrkA, which exhibits tumour suppressor function in NB and associates with better prognosis, TrkAIII expression associates with advanced stage metastatic disease, post therapeutic relapse and poor prognosis. TrkAIII also induces malignant transformation of NIH3T3 cells and exhibits oncogenic activity in NB models [1, 19–22], confirming its oncogenic nature and suggesting that patients with tumours that express TrkAIII may benefit from Trk-inhibitory therapy.

## TrkAIII detection and association with human malignancies

**Fig. 1:**
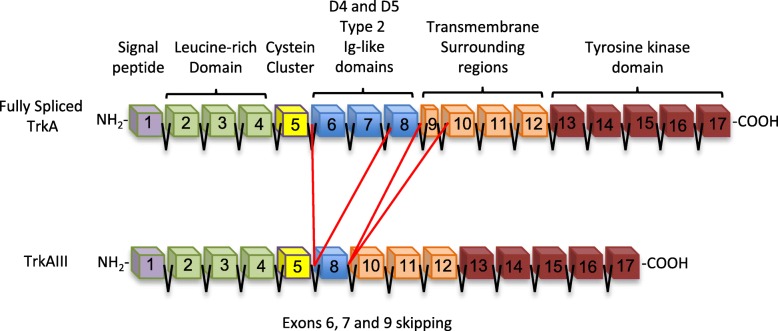
Fully spliced TrkA and alternatively spliced TrkAIII exon structure. Schematic representation of the exon structures of fully spliced TrkA, with associated receptor domains, and alternatively spliced TrkAIII, exhibiting exons 6, 7 and 9 skipping and deletion of the D4 Ig-like domain

**Fig. 2:**
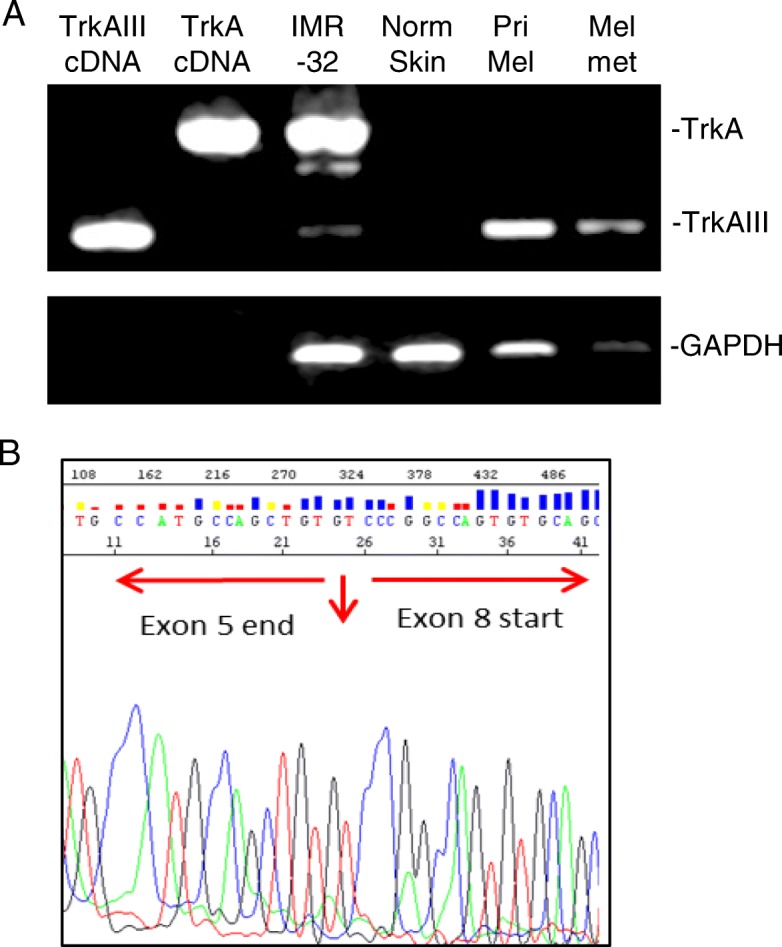
TrkAIII is expressed in primary and secondary to metastatic melanomas. **a**) Representative RT-PCR agarose gel, demonstrating exclusive TrkAIII expression in a single primary melanoma (Pri Mel) and single melanoma metastasis (Mel Met) samples compared to predominant fully spliced TrkA expression in IMR32 NB cells and lack of TrkA expression in a sample of normal human skin plus TrkAIII and TrkA cDNA RT-PCR product controls and GAPDH, as a loading control. **b**) Representative TrkAIII exon 5/8 splice junction sequence from a melanoma metastasis

TrkAIII was originally detected as an unexpected TrkA RT-PCR product in primary human NBs and full length TrkAIII cDNA was subsequently cloned from human SH-SY5Y NB cells. TrkAIII represents a novel alternative *TrkA* splice variant that exhibits skipping of exons 6, 7 and 9, resulting in deletion of the receptor extracellular D4 Ig-like domain and several N-glycosylation sites (Fig. 1) [19]. In human NBs, TrkAIII expression associates with advanced-stage metastatic disease, post-therapeutic relapse and worse prognosis, whereas expression of fully spliced TrkA associates with better prognosis, spontaneous regression and enhanced event-free survival [1, 19–22]. In addition to NBs, human glioblastoma multiforme tumours also express TrkAIII, including an EGFR and EGFRvIII negative subpopulation, suggesting that TrkAIII represents a potential oncogenic alternative to EGFR and EGFRvIII oncogenes in this tumour type [23]. Recently, we have also detected alternative TrkAIII splicing in cutaneous melanomas, with sequence-verified evidence for exclusive TrkAIII expression in individual primary and metastatic tumours. This novel, not previously published data, supports a potential role for TrkAIII in melanoma pathogenesis and progression (manuscript in preparation) (Fig. 2). TrkAIII expression in melanoma may help to explain the reported association between malignant melanoma and intracellular TrkA activation, considering that the expression of fully spliced TrkA exhibits paradoxical tumour suppressing activity in melanoma cells [24]. TrkAIII expression in melanoma, furthermore, may also combine with *TrkA* gene amplification, reported in up to 50% of tumours [25]. In tumour cell lines, both constitutive and stress-regulated TrkAIII expression has been detected in numerous NB cell lines, PC-3 prostate cancer cells, PC-12 pheochromocytoma cells, U251 glioma cells and Jurkat T cell leukaemia cells [19, 22, 26, 27].

Routine RT-PCR detection of fully spliced TrkA and alternatively spliced TrkAIII, utilising exon 8 and exon 5 primers sets [19], has been improved by adding TrkAIII-specific and exons 6/7-specific primer sets that discriminates between TrkAIII and TrkA cDNAs containing exon 6/7 sequence. The TrkAIII-specific primer set: forward primer 5’-TGC gCA ATG CCA GCT GTG TaC CgG tCa-3′ (novel exon 5–8 splice junction), bears point mutations (lower case) and reverse primer 5′-AGT ATT GTG GGT TCT CGA TGA TGT-3′ (exon 12) that abrogate amplification of exons 6/7-containing TrkA cDNAs, without compromising TrkAIII cDNA amplification. The 6/7-containing TrkA-specific primer set: forward primer 5′-TGA AGG TCC AGG TGC CCA AT -3′ and reverse primer 5′-TTG ACC TGA ACA GAG ACC TCT GC-3′, amplifies sequences contained within exons 6 and 7 but does not amplify TrkAIII cDNA.

True assessment of TrkAIII expression and function in human malignancies, however, suffers from a lack of TrkAIII-specific antibodies, making correlation between TrkAIII mRNA and protein expression difficult to assess in tumour samples. Furthermore, difficulty in obtaining TrkAIII expressing NB cells from TrkAIII expressing NB samples and the fact that alternative TrkAIII splicing is stress-regulated and likely, therefore, to be lost in vitro, as reported for the expression of EGFRvIII splice variant oncogene [28], has led to the predominant use of transfected cell lines to study TrkAIII oncogenic behaviour, raising some concerns. Development of a TrkAIII-specific antibody will, therefore, be critical for routine immunohistochemical detection of TrkAIII protein expression in order to identify tumours that express TrkAIII mRNA but not TrkAIII protein, which would not be expected to respond to Trk inhibitory therapy [18].

## Oncogenic alternative TrkAIII splicing: A De-regulated physiological stress-protection mechanism and tumour-promoting switch

Stress-regulated alterative TrkAIII splicing is not only exhibited by neural and neural crest related tumour cells but also by normal human neural stem cells and neural crest-derived progenitors but not differentiated counterparts. TrkAIII is also expressed in normal embryonic and post-natal thymuses and by thymic epithelial cell and thymocyte subpopulations, suggesting that alternative TrkAIII splicing represents a developmentally-regulated physiological mechanism that is relatively restricted to neural-related stem/progenitor cells, the thymus and thymocytes, during development [19, 27, 29].

How this is subverted into an oncogenic mechanism in neural/neural crest-related cancers is not fully understood. A direct transforming role for TrkAIII would have to involve pre-existing changes in neuroblasts, such as functional pRB and/or p53 inactivation, implicated in single-oncogene transformation of NIH3T3 cells [30], induced by TrkAIII [19]. This could be enhanced by stress within the microenvironment, providing a way to transform tumour-suppressing TrkA signals into oncogenic signals from TrkAIII [19, 29]. On the other hand, TrkAIII expression by normal non-altered neuroblasts would be expected to be temporary, reversible and insufficient to induce transformation. TrkAIII activation in neuroblasts, furthermore, would require overcoming intracellular spontaneous ligand-independent activation thresholds, which may differ in different intracellular compartments. TrkAIII spontaneous activation potential, already enhanced by deletion of the D4 spontaneous activation-prevention domain [19, 31], could be increased by constant constitutive expression and/or by overexpression, resulting from *TrkA* gene amplification. In support of the latter, *TrkA* gene amplification has been detected in a wide a range of human cancers, including melanomas [13, 25], which express TrkAIII (Fig. 2) (manuscript in preparation). Spontaneous TrkAIII activation could also result from down-regulating the expression and activity of TrkA de-phosphorylating PTPases, such as Shp1 and PTP1B [19, 32], in a manner analogous to PTP1B regulation of the NB-associated oncogene Alk [33, 34], which may also influence potential interactions between Alk and TrkAIII [35].

Alternatively, neuroblasts transformed by oncogenes, such as Alk [33], may utilise stress-regulated alternative TrkAIII splicing to augment survival during tumour initiation and early expansion, with de-regulation into a fully-fledged oncogenic mechanism occurring at a later stage. In this scenario, alternative TrkAIII splicing could be reversed, increasing potential for spontaneous regression and post therapeutic event-free survival, both of which associate with NB expression of fully spliced TrkA [1, 2, 20, 36].

**Fig. 3:**
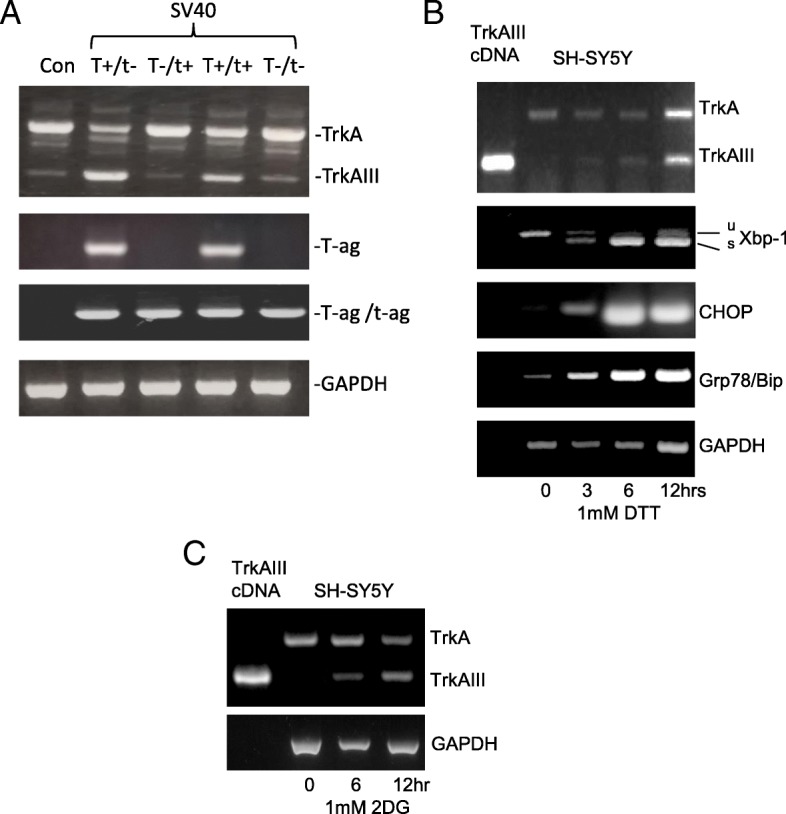
SV40 large T antigen, DTT and 2-DG promote alternative TrkAIII splicing in NB cells. **a**) Representative RT-PCR agarose gel demonstrating the promotion of sequence verified alternative TrkAIII expression in SH-SY5Y NB cells, following transient transfection with plasmids pw2dl expressing SV40 large T-ag alone (T+/t-), pw2t expressing SV40 small tag alone (T−/t+), pw2 expressing both large T-ag and small t-ag (T+/t+) and negative control pw101 expressing only mutation inactivated small t-ag (T−/t-) compared to non-transfected control (Con). Large T-ag expression was confirmed by RT PCR amplification using T-ag specific primers whilst small tag expression was confirmed by deduction using a primer set that generates an identical fragment form both large T-ag and small tag cDNAs. **b**) Representative RT-PCR agarose gels demonstrating the promotion of alternative TrkAIII splicing in SH-SY5Y cells treated with 1 mM DTT for 3–12 h plus DTT-induced Xbp-1 splicing, CHOP and Grp78/Bip expression compared to GAPDH levels. **c**) Representative RT-PCR demonstrating induction of alternative TrkAIII splicing in SH-SY5Y cells treated for 6 and 12 h with the glucose deprivation mimic 2 deoxy-glucose (1 mM 2-DG) plus TrkAIII cDNA and GAPDH RT PCR controls

TrkAIII association with advanced stage metastatic disease, post-therapeutic relapse and worse prognosis [19–21], however, suggests that a significant proportion of NBs exhibit more permanent alternative TrkAIII splicing. This could result from *TrkA* splice site mutations that promote exon 6–7 skipping, although to date splice site mutations have not been detected in either TrkAIII expressing NBs [19] or melanomas (unpublished observations). Alternatively, we have also found that SV40 large T-Antigen promotes alternative TrkAIII splicing in SH-SY5Y NB cells transiently transfected with the plasmids expressing different combinations of SV40 large T and small t antigens (pw2dl: SV40 large T-ag alone, T+/t-; pw2t: small t-ag alone, T−/t+; pw2: large T-ag and small t-ag, T+/t+; and a negative control pw101: mutation-inactivated small t-ag without T-ag, T−/t-) [37] (Fig. 3a). This novel, not previously published data suggests that polyoma virus infection, which has been associated with increased risk of NB and identified in NB samples (reviewed in [38]), may promote alternative TrkAIII splicing in neuroblasts. Furthermore, SV40 T-ag expression induces NB in mice [39]. Alternative TrkAIII splicing in NB cells is also promoted by cobalt salts [19], suggesting that heavy metals that induce oxidative stress may also de-regulate TrkA splicing. Finally, mechanisms that de-regulate splice factor expression and/or activity to induce alternative splicing, such as the DNA damage-, ER-stress and nutrient-stress responses [40–42], may also de-regulate alternative TrkAIII splicing. In support of this, novel not previously published observations indicate that treatment of SH-SY5Y NB cells with ER stress inducing agent dithiothreitol (DTT) or with with the non-metabolisable glucose-deprivation mimic 2-deoxy-glucose (2DG) promote alternative TrkAIII splicing (Fig. 3b and c).

## TrkAIII triggers a variety of pro-tumorigenic mechanisms

TrkAIII induces malignant transformation of NIH 3 T3 cells [19] in a single oncogene transformation mechanism, characterising TrkAIII as the pathological equivalent of an engineered oncogenic TrkA D4 Ig-like domain deletion-mutant, previously described [31]. In transfected NB cells, TrkAIII but not fully spliced TrkA augments xenograft tumorigenicity in vitro and in vivo*,* comparable to TrkT3 oncogene, and also augments osteolytic bone metastasis and non-bone metastasis formation in a nude mouse xenograft metastasis model, confirming that TrkAIII promotes both primary tumorigenicity and metastatic capacity. TrkAIII promotion of osteolytic bone metastasis associates with osteoclast differentiation and expression of the osteoclast-activating cytokine RANKL [19, 23, 29]. The relatively hypoxic bone metastatic microenvironment, furthermore, provides a suitable environment to promote and maintain alternative TrkAIII splicing, suggesting that TrkAIII promotion of bone metastasis may represent a novel organ specific metastatic mechanism, supporting Paget’s “Seed and Soil” hypothesis for organ specific metastasis [23].

### TrkAIII signaling

In NB transfectants, NGF activation of fully spliced TrkA receptors results in receptor tyrosine phosphorylation and signaling through both PI3K/Akt/NF-κB and RAS/MAPK pathways, resulting in neuronal differentiation, characterised by inhibition of proliferation and neuritogenesis [1, 19]. In contrast, TrkAIII transfectants do not respond to extracellular NGF but exhibit spontaneous intracellular ligand-independent TrkAIII activation that results in chronic signaling through the PI3K/Akt/NF-κB but not RAS/MAPK pathway and continuous proliferation in the absence of neuritogenesis [19]. This implicates RAS/MAPK signaling in the NB tumour-suppressing effects of NGF-activated TrkA receptors and PI3K/Akt/NF-κB without RAS/MAPK signaling in the diametrically opposed oncogenic activity of TrkAIII, which for some reason is unable to activate the RAS/MAPK pathway, despite binding Grb-2 and Frs-2 adapter proteins involved in RAS/MAPK activation [43].

### TrkAIII pro-Angiogenic PI3K/Akt/NF-кB signaling

In contrast to fully spliced TrkA expression, which reduces NB xenograft tumorigenicity, tumour-associated angiogenesis and angiogenesis factor expression by NB cells [44, 45], xenograft tumours formed by TrkAIII transfectants are more vascular than tumours formed by control or fully spliced TrkA transfectants [19], consistent with a more angiogenic phenotype. In support of this, TrkAIII transfectants express elevated levels of the angiogenesis and metastasis-associated factors VEGF and MMP-9 and reduced levels of the angiogenesis inhibitors thrombospondin (Tsp)-1 and TIMP-3 compared to control and TrkA transfectants [19]. The MMP-9/VEGF/Tsp1 equilibrium is considered critical for tumour angiogenesis, since MMP-9 activates VEGF and Tsp-1 inhibits both MMP-9 activation and VEGF activity [46, 47], and TIMP-3 is a potent angiogenesis inhibitor [48]. This pro-angiogenic equilibrium is regulated by PI3K in TrkAIII transfectants and is reversed by the PI3K inhibitor LY294002, which reduces VEGF and MMP9 expression and enhances Tsp-1 expression in TrkAIII transfectants [19].

### TrkAIII pro-survival PI3K/AKT/NF-кB signaling

PI3K/Akt/NF-кB signaling is also important for cell survival, and TrkAIII transfectants exhibit enhanced resistance to genotoxic- (cisplatin), oxidative- (paraquat, rotenone and LY83583) and ER-stress (DTT, A23187 and thapsigargin) [19, 32, 49]. Increased stress-resistance exhibited by TrkAIII transfectants associates with elevated expression and mitochondrial localisation of anti-apoptotic Bcl-2, Bcl-xL and Mcl-1 proteins and also with enhanced expression of the mitochondrial anti-oxidant SOD-2 [32, 49]. Trk tyrosine kinase inhibitors (K252a, CEP-701 and Gö6976), PI3K inhibitor (LY294002) and NF-κB inhibitors (dn-NF-кB and PDTC), all reduce Bcl-2, Bcl-xL and SOD2 expression in TrkAIII transfectants and increase sensitivity to genotoxic-, oxidative- and ER-stress induced death, confirming dependence upon TrkAIII tyrosine kinase, PI3K and NF-кB activity for survival [19, 32, 49].

Increased mitochondrial SOD2 expression and activity in TrkAIII transfectants attenuates constitutive and induced (paraquat, rotenone and LY83583) mitochondrial free-radical ROS production and increases resistance to agents that induce mitochondrial free radical-mediated death, identifying a novel TrkAIII/SOD2 mitochondrial protection axis [49].

### TrkAIII mislocalization as a mechanism for oncogenic activity

**Fig. 4:**
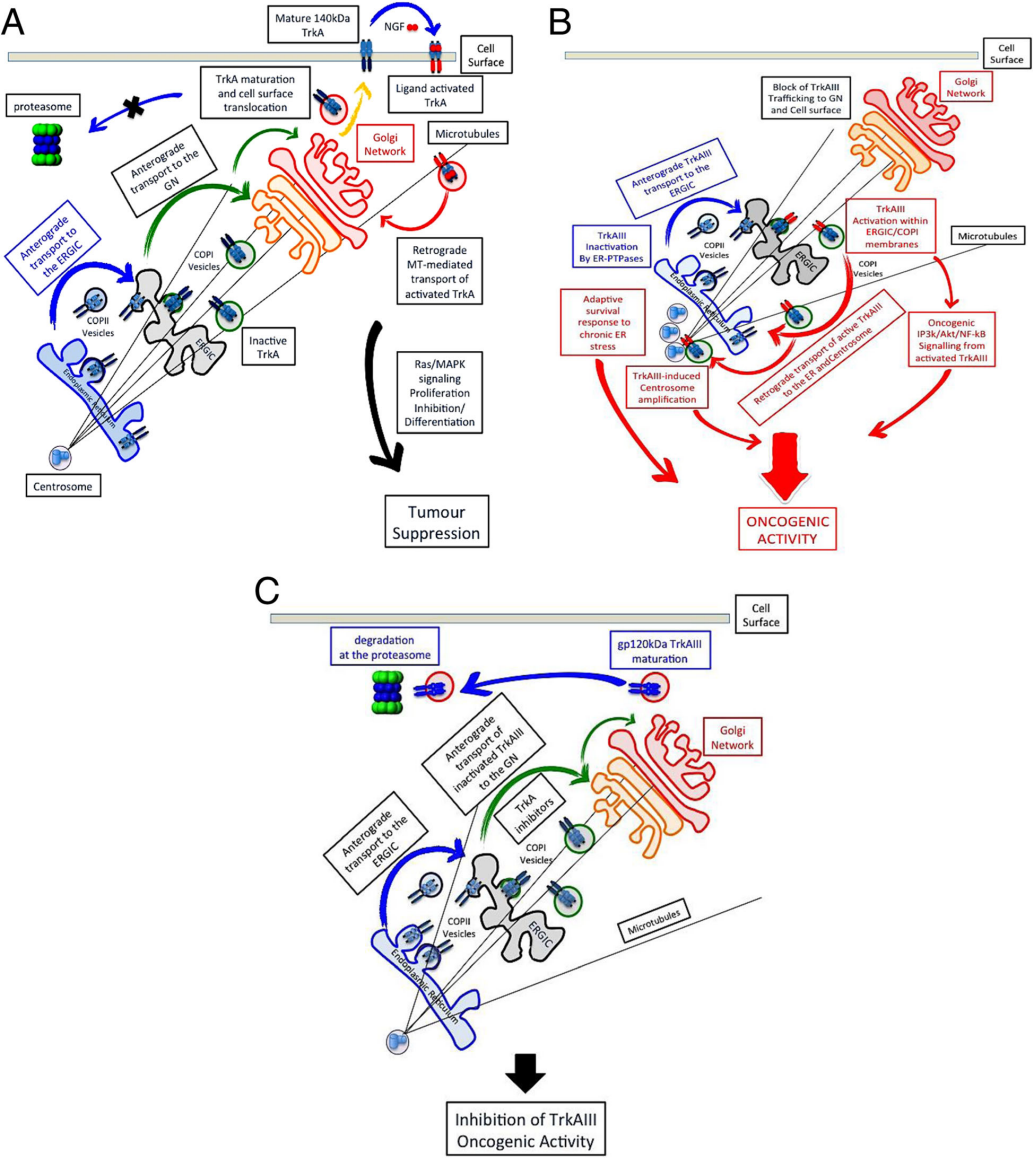
TrkAIII recycles between the ERGIC and ER compartments. Schematic representation of: **a**) fully spliced TrkA receptor trafficking from intracellular to cell surface compartments, characterised by anterograde transport of inactive immature TrkA receptors from the ER to the ERGIC and GN, where they are matured prior to being transported to the cell surface plus the fate of neurotrophin-activated cell surface TrkA receptors that exhibit motor protein-dependent MT minus-end directed retrograde transport to the GN, associated with the activation of tumour suppressing Ras/MAPK signaling. **b**) TrkAIII intracellular trafficking, characterised by anterograde transport of inactive immature TrkAIII from the ER to the ERGIC, spontaneous activation within ERGOC/COPI membranes that blocks anterograde transport to the GN and promotes MT minus-end directed retrograde transport of active TrkAIII back to the ER and centrosome, setting up self perpetual TrkAIII recycling between the ER and ERGIC, partial UPR activation, PIP3K/Akt/NF-κB pro-survival and pro-angiogenic signaling, centrosome amplification, chromosome instability, formation of micronuclei and increased MTOC activity. **c**) Altered TrkAIII trafficking in the presence of Trk tyrosine kinase inhibitor, characterised by the uninhibited anterograde transport of inactive TrkAIII from the ER to ERGIC and GN, resulting in GN-associated gp120kDa TrkAIII maturation, degradation of mature TrkAIII at the proteasome and the inhibition of TrkAIII oncogenic activity

TrkAIII intracellular re-localisation and oncogenic behaviour supports the growing concept that mislocalization of TrkA-derived oncogenes underpins oncogenic signaling [3, 7, 19, 26, 32, 40, 50]. In contrast to fully-spliced cell surface TrkA receptors that associate with caveolin in low density membrane fractions [23] and exhibit tumour suppressing activity in NB [1, 19], TrkAIII receptors do not associate with caveolin in low density membrane fractions [23] and re-localise to intracellular pre-Golgi membranes, in which they exhibits spontaneous activation and self-perpetual recycling between the ER and ERGIC-COPI compartments [19, 40, 50]. Pulse chase and membrane purification analyses clearly demonstrate that TrkAIII exits the ER and arrives at the ERGIC [40, 50] but fails to exhibit anterograde transport to the Golgi Network (GN) [40]. Instead, TrkAIII exhibits spontaneous activation within ERGIC-COPI membranes, which results in microtubule (MT)-dependent, MT-minus end retrograde transport from the ERGIC/COPI compartment back to the ER, where TrkAIII is inactivated prior to returning once more to the ERGIC [39, 50]. This sets up self-perpetual ER and ERGIC-COPI recycling that not only ensures sufficient accumulation of TrkAIII within the ER to induce partial UPR activation but also sufficient accumulation of TrkAIII to overcome the spontaneous activation threshold within the ERGIC/COPI compartment and retrograde transport of TrkAIII back to the centrosome, where it complexes with both γ- and α-tubulin [26, 51]. This behaviour is reminiscent of motor protein MT minus-end directed retrograde transport of activated cell surface TrkA receptors that translocate from axon terminals along axons to the neuronal cell body [52]. Furthermore, the inhibition of TrkAIII anterograde transport to the GN prevents GN-associated TrkAIII maturation. This can be overcome by Trk tyrosine kinase inhibitors (CEP-701, Gö6976 and GW441756), which promote anterograde TrkAIII transport to the GN, resulting in gp120kDa TrkAIII maturation and degradation at the proteasome [40]. Degradation of inactivated mature gp120kDa TrkAIII at the proteasome, implicates the amino acid sequence deleted from TrkAIII in protecting fully spliced TrkA receptors from proteasome degradation as they translocate to the cell surface, as cell surface expression of mature gp120kDa TrkAIII is only detected under TrkAIII and proteasome inhibitory conditions (Schematized in Fig. 4) [40, 53, 54].

Spontaneous TrkAIII activation within ERGIC/COPI membranes, confirmed by co-localisation with ERGIC and COPI markers in purified membrane fractions and also by confocal microscopy, is prevented by the COPI vesicle ARF-inhibitor Brefeldin A and indicates that the ERGIC/COPI membrane compartment is more permissive for TrkAIII activation [40]. This adds to reports that the COPII membrane compartment is permissive for Trk-oncogene activation and that immature TrkA receptors, blocked in anterograde transport, are aberrantly activated within ERGIC membranes [55, 56]. It appears, therefore, that ERGIC/COP membranes exhibit a lower threshold for spontaneous TrkAIII activation than either ER or GN compartments [19, 31]. This lowered threshold is likely not only to depend upon changes in TrkAIII interactions within a vesicular context but also by lower levels of TrkAIII dephosphorylating PTPase within this membrane compartment. Activation within the ERGIC/COPI compartment may also explain why TrkAIII does not activate the RAS/MAPK pathway, as the ERGIC/COPI compartment would be dissociated from RAS/MAPK components in the GN and cell surface compartments [57]. In support of this, immature TrkA receptors blocked in anterograde transport and activated within the ERGIC compartment do not activate RAS/MAPK signaling [56], supporting the role of RAS/MAPK signaling in the diametrically opposed tumour suppressing and oncogenic behaviours exhibited by TrkA and TrkAIII, respectively [19, 49, 51].

### TrkAIII, ER stress and the unfolded protein response (UPR)

**Fig. 5:**
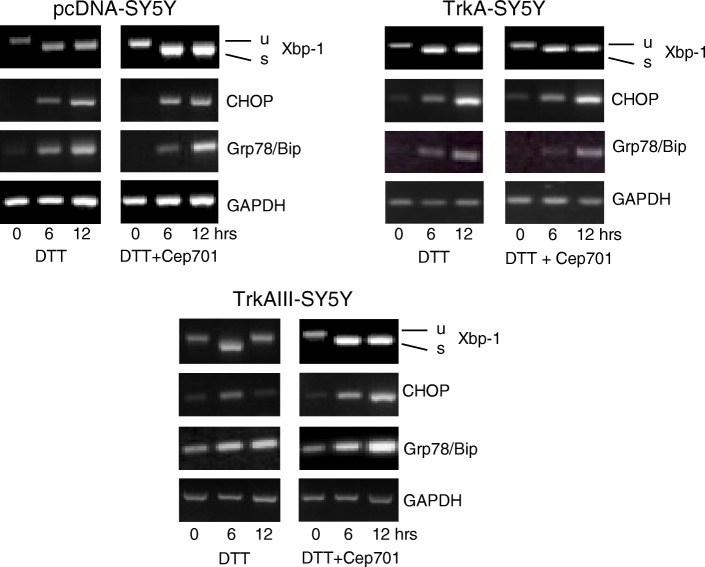
TrkAIII preconditions NB cells to better survive and resolve acute ER stress. Representative RT-PCR agarose gels comparing DTT induced Xbp-1 splicing, CHOP and Grp78/Bip expression in control, fully spliced TrkA and TrkAIII SH-SY5Y transfectants, demonstrating that TrkAIII transfectants exhibit complete attenuation of DTT-induced Xbp-1 splicing and CHOP but not Grp78/Bip expression within 12 h, not observed in either control or TrkA transfectants plus the prevention of this effect in TrkAIII transfectants pre-incubated for 1 h with 100 nM CEP-701 prior to DTT treatment

In addition to pro-survival and pro-angiogenic PI3K/Akt/NF-кB signaling, TrkAIII expression also results in partial activation of a survival-adapted UPR, characterised by ATF6 but not Ire1a/Xbp-1 activation, and increased expression of the UPR chaperone Grp78/Bip and the apoptosis inhibitor Mcl-1 [32, 40, 50, 58]. In TrkAIII transfectants, ER-associated TrkAIII is complexed with Grp78/Bip, confirming that TrkAIII has difficulty in satisfying ER quality control and helping to explain partial UPR activation [50]. The lack of constitutive Ire1a/Xbp-1 activation does not depend upon a defect in Ire1a/Xbp-1, as agents that induce acute ER stress readily activate Ire1a/Xbp-1 in TrkAIII transfectants (Figs. 3 and 5) [50]. Furthermore, Trk tyrosine kinase inhibitors do not induce Xbp-1 splicing in TrkAIII transfectants, indicating that lack of Ire1a/Xbp-1 activation does not depend directly upon TrkAIII tyrosine kinase activity. This response, therefore, appears to represent a survival UPR adaptation to chronic sub-lethal ER stress, adding a novel “non-addiction” mechanism to TrkAIII oncogenic activity. This partial UPR, pre-conditions TrkAIII transfectants to better survive and resolve acute ER-stress, enhancing resistance to agents that induce ER stress compared to control and TrkA transfectants [32]. Furthermore, novel not previously published observations have detected a difference in the UPR induced by dithiothreitol (DTT) in control and TrkA transfectants, characterised by induction of Xbp-1 splicing and expression of the apoptosis promoter CHOP that is maintained throughout the 12-h time course, when compared to TrkAIII transfectants in which DTT-induced Xbp-1 splicing and CHOP expression are rapidly attenuated, returning to normal levels by 12 h (Fig. 5), in association with enhanced survival [32]. The Trk inhibitor CEP-701 (100 nM) prolongs the UPR in TrkAIII transfectants (Fig. 5), in association with increased death [32]. Since, CHOP expression is regulated by PERK/ATF-4 and not by Ire1a/Xbp-1 [59–61], the attenuation of DTT-induced Xbp-1 splicing and CHOP expression in TrkAIII transfectants must reflect the inhibition of both PERK/ATF-4 and Ire1a activity, indicating a more rapid resolution of ER stress. This supports a role for constitutive TrkAIII tyrosine kinase activity combined with pre-existing TrkAIII-dependent ATF6 activation [50] and elevated Grp78/Bip, Bcl-2 protein and SOD2 expression [32, 58], in cellular pre-conditioning to better survive and resolve episodes of acute ER stress.

### TrkAIII, centrosome amplification, chromosomal instability and MTOC activity

Retrograde MT minus-end transport not only results in TrkAIII transport back to the ER but also translocation of activated TrkAIII to the centrosome, where it localises in complexes with γ- and α-tubulin [26, 51]. TrkAIII, at the centrosome, tyrosine phosphorylates centrosome components, including Plk4, leading to de-regulation of centrosome duplication, resulting in centrosome amplification, increased multi-polar mitotic spindle formation, polyploidy, aneuploidy and the formation of micronuclei [26, 51]. Micronuclei formation has been reported to drive metastasis through the cytosolic cGAS-STING DNA response [62], suggesting that TrkAIII activity at the centrosome resulting in micronuclei formation may represent an important metastasis driver mechanism, in addition to chromosomal instability. TrkAIII at the centrosome also tyrosine phosphorylates α-tubulin and promotes MT polymerisation, augmenting MTOC activity during interphase. This results in the formation of short concentrated MT-arrays during interphase that promote nuclear anaplasia and help maintain NB cells in an undifferentiated state [51].

Therefore, retrograde TrkAIII transport and recycling between the ER and ERGIC/COPI compartments in addition to reinforcing ERGIC/COPI-associated TrkAIII activation, pro-survival and pro-angiogenic PI3K/Akt/NF-кB signaling and a UPR survival-adaption also results in accumulation at the centrosome, promoting centrosome amplification, chromosome instability, micronuclei formation and MTOC activity, increasing metastatic potential and hindering differentiation.

### TrkAIII and sister chromatid exchange

In addition to increasing chromosomal instability, TrkAIII transfectants also exhibit increased levels of sister chromatid exchanges, suggesting that TrkAIII may also promote replication-stress either by elevating levels of homologous recombination and/or the burden of single strand DNA breaks [26]. As for other oncogenes, this could be achieved by altering the expression of genes involved in licensing replication origins and/or replication fork elongation, de-regulating DNA replication dynamics and promoting genomic instability to drive tumorigenesis [63]. Furthermore, TrkAIII is also detected in nuclear extracts [23], suggesting that TrkAIII may also play a role within the nucleus.

### TrkAIII, differentiation and tumour cell staminality

TrkA expression is a prerequisite for NB cell differentiation and NB regression, via RAS/MAPK-dependent inhibition of proliferation and induction of neuritogenesis [1, 19, 36]. TrkAIII transfectants do not respond to exogenous NGF and spontaneous intracellular TrkAIII activation does not activate RAS/MAPK signaling, resulting in continuous proliferation in the absence of neuritogenesis [19]. Furthermore, TrkAIII expression also inhibits the differentiation-inducing effects of NGF-activated TrkA signaling by preventing RAS/MAPK activation, characterising TrkAIII as a potential pivotal regulator of NB cell differentiation in the presence of neurotrophins [19, 23]. TrkAIII transfectants also exhibit elevated expression of the NB tumour stem cell-markers CD117, SOX2, Nestin and Nanog, indicating that TrkAIII not only blocks NGF/TrkA-induced NB cell differentiation but also promotes a more NB tumour stem cell-like phenotype. This provides an additional explanation for the reported association between TrkAIII expression and advanced stage metastatic disease, post-therapeutic relapse and worse prognosis in NB, all of which associate with a more stem cell like phenotype [19–21].

### TrkAIII mitochondrial translocation and induction of the “Warburg” effect

**Fig. 6:**
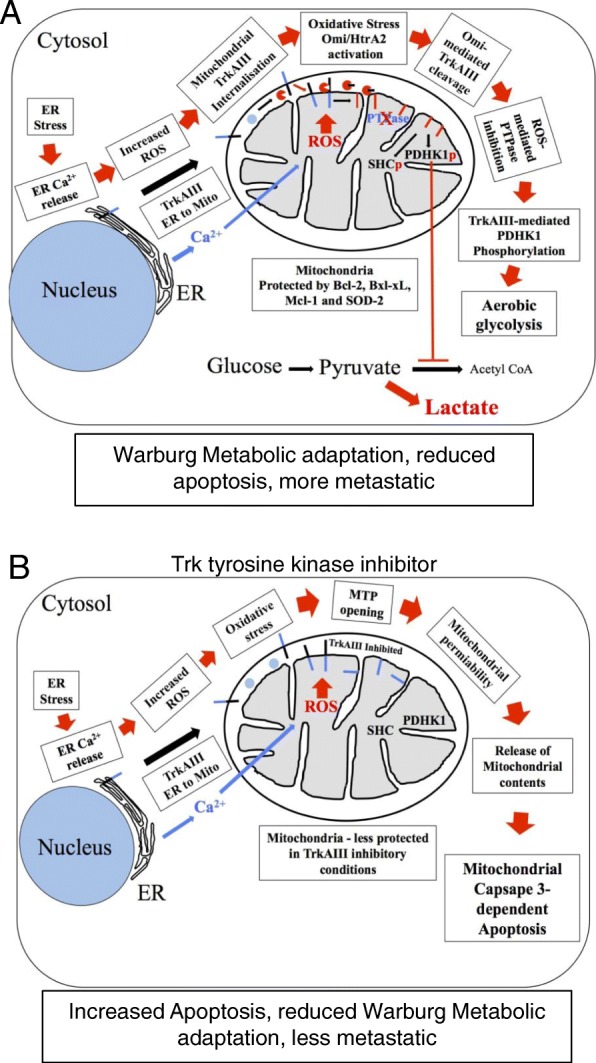
ER-stress promotes mitochondrial TrkAIII translocation, activation and the Warburg effect. Schematic representation of: **a**) ER stress induced translocation of MAM-associated TrkAIII into mitochondrial inner membranes, resulting in Omi/HtrA2-dependent TrkAIII cleavage activation, PDHK1 tyrosine phosphorylation and aerobic glycolytic “Warburg” metabolic adaptation, involving ER Ca2+ release and uptake by mitochondria and increased ROS production, within the context of mitochondria protected against apoptosis by increased Bcl-2 proteins and SOD2 expression, and **b**) The effect of Trk tyrosine kinase inhibitor on ER stress-induced mitochondrial TrkAIII translocation and cleavage but inactivation, leading to reduced Warburg metabolic adaptation and increased apoptosis, associated with reduced Bcl-2 protein and SOD2 expression

Continuous TrkAIII recycling between ER and ERGIC membranes [40] also leads to the re-localisation of TrkAIII to specialised membranes sites (MAMs) that link the ER to the mitochondria, resulting in TrkAIII association with outer mitochondrial membranes (OMM) [32]. Under normal conditions, OMM-associated TrkAIII is not constitutively tyrosine phosphorylated, indicating that the MAM-mitochondrial environment has a high threshold for spontaneous TrkAIII activation. However, under conditions of ER stress, induced by DTT, thapsigargin or A23187 calcium ionophore, OMM-associated TrkAIII is rapidly internalised into inner mitochondrial membranes (IMMs), where it is subjected to Omi/HtrA2-dependent cleavage-activation to 45-48 kDa CT active fragments, in mitochondrial matrix orientation [32]. Since TrkAIII does not posses a typical mitochondrial translocation sequence, mitochondrial importation under conditions of ER stress may be the result of the stress-regulated mitochondrial import mechanism for the uptake and degradation of damaged proteins that forms part of the mitochondrial UPR [64]. Activation of IMM-associated TrkAIII under conditions of ER stress is facilitated by ROS/Ca^2+^ interplay, providing conditions necessary for activation of mitochondrial Omi/HtrA2, which is responsible for eliminating the remaining N-terminal spontaneous activation-preventing sequences from TrkAIII, which together with ROS inactivation of mitochondrial PTPases facilitates the spontaneous activation of IMM-associated cleaved TrkAIII. The activation of IMM-associated TrkAIII results in the tyrosine phosphorylation of mitochondrial proteins including pyruvate dehydrogenase kinase-1, an inhibitor of the pyruvate dehydrogenase complex, resulting in a metabolic switch to aerobic glycolysis [32]. This identifies TrkAIII as a novel communicator of ER-stress to the mitochondria that results in metabolic adaptation, providing a mechanism through which ER-stress helps to maintain the metastasis-promoting “Warburg” metabolic effect in TrkAIII expressing tumour cells [64–67] (Schematized in Fig. 6).

## TrkAIII and the hallmarks of cancer

**Fig. 7:**
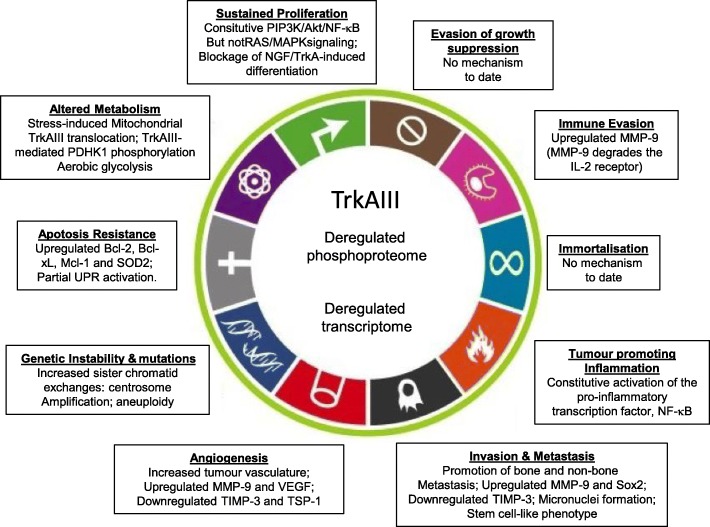
TrkAIII influences the majority of Cancer Hallmarks. Schematic representation of TrkAIII deregulation of the phospho-proteome and transcriptome and how TrkAIII influences individual “Hallmarks of Cancer”

Cancer is characterised by 10 hallmarks, providing order to the many mechanisms involved in malignant transformation [68]. Our summary of the many ways TrkAIII influences the oncogenic process, clearly demonstrates the aberrant TrkAIII expression and activation regulate the majority, if not all of the hallmarks of cancer. Although TrkAIII influence on “Evasion of Growth Suppression” and “Immortalization” hallmarks, have yet to be described, TrkAIII influences the hallmarks of “Immune Evasion” by up-regulating MMP-9, cFLIP and Mcl-1 expression [19, 32, 58]; “Tumour-associated Inflammation” through constitutive activation of the pro-inflammatory transcription factor NF-кB [19]; “Invasion and Metastasis” by up-regulating MMP-9 and down-regulating TIMP-3 expression [19]; “Angiogenesis” through PIP3K-mediated upregulation of MMP-9 and VEGF expression, down-regulation of TIMP-3 and thrombospondin-1 expression [19] and promotion of micronuclei formation [26]; “Genetic Instability” by increasing sister chromatid exchange and inducing centrosome amplification, resulting in aneuploidy [26]; “Apoptosis Resistance” by up-regulating Bcl2, Bcl-xL, Mcl-1 and SOD-2 expression and partial activation of a survival-adapted UPR [32, 58, 49]; “Metabolism” through ER stress-induced mitochondrial translocation, activation and PDHK1 tyrosine phosphorylation, resulting in a switch to aerobic glycolysis [40]; and “Sustained Proliferation” by activating chronic PI3K/Akt in the absence RAS/MAPK signaling, inhibiting pro-differentiation signaling from NGF-activated TrkA and maintaining a tumour stem cell-like phenotype [19, 49] (Schematized in Fig. 7).

## Potential therapeutic approaches for TrkAIII-driven cancers

### Small molecule Trk tyrosine kinase inhibitors

Adult and childhood cancers driven by novel TrkA-fusion oncogenes exhibit profound and long-lived responses to the Trk inhibitor Larotrectinib [18]. This suggests that cancers driven by TrkAIII may also represent good candidates for small molecule therapeutic Trk tyrosine kinase inhibitors, such as Larotrectinib, Entrectinib, Cabozantinib, Merestinib, TRS-011, DS-6051b, MGCD516, PLX7486 and DCC-2710, currently employed in ongoing clinical trials for cancers driven by novel *Trk*-fusions or altered Trk activity ([11, 12] and reviewed in [17]) or novel Trk inhibitors in development, for which patents have been issued [69]. The therapeutic efficacy of small molecule Trk inhibitors could also be enhanced if combined with cancer drugs that induce genotoxic-, oxidative- or ER-stress, which would be expected to augment tumour cell killing, upon inhibition of TrkAIII-dependent survival signaling.

### Inhibitors of TrkAIII expression

TrkAIII expression can be directly inhibited by the TrkAIII-specific PNA inhibitor (KKAA)_4_-GGCCGGGACACA, or equivalent TrkAIII-specific inhibitory siRNAs, which decrease TrkAIII expression in TrkAIII transfectants and enhance sensitivity to cancer drugs [26, 50]. As stated above, efficacy of TrkAIII expression inhibitors could be enhanced if combined with cancer drugs that induce genotoxic-, oxidative- or ER-stress, following a significant inhibition of TrkAIII expression and TrkAIII-dependent pro-survival signaling.

### Reversing alternative TrkAIII splicing

Considering the NB tumour suppressing activity of fully spliced TrkA, reversal of alternative TrkAIII splicing back to fully spliced TrkA expression may represent an important therapeutic goal. This could be achieved using new generation lentiviral vectors engineered to express TrkAIII inhibitory siRNAs and the fully spliced TrkA coding sequence, mutated in wobble sites to prevent off-target inhibition by TrkAIII-specific siRNA, without altering the TrkA amino acid sequence. This approach would block TrkAIII oncogenic activity and reinstate TrkA tumour-suppressing activity, with potential to slow advanced disease progression, enhance chemotherapeutic-sensitivity and increase the potential for spontaneous regression and post-therapeutic event-free survival, all of which associate with fully spliced TrkA expression [1, 20, 36].

### Inhibition of PI3K/AKT/NF-кB signaling

TrkAIII promotion of pro-survival and pro-angiogenic PI3K/Akt/NF-кB signaling exhibited by TrkAIII transfectants suggests that inhibitors of PI3K/Akt/NF-кB signaling could significantly slow tumour progressions, reduce tumour-associated angiogenesis and enhance chemotherapeutic-sensitivity. This could be achieved either by plant-derived PI3K inhibitors (reviewed in [70]), synthetic pan- and isoform-specific PI3K inhibitors and Akt inhibitors (reviewed in [71]) or by the > 700 small molecule upstream NF-кB inhibitors of IKK activity, IкB phosphorylation and IкB degradation, inhibitors of NF-кB transactivation, proteasome activity and NF-кB inhibitory antioxidants (reviewed in [72]).

### Targeting the UPR

TrkAIII induces partial activation of a survival-adapted UPR, which preconditions cells to better survive and resolve acute episodes of ER stress, in an important “non-oncogene addiction” mechanism. Agents (siRNA, PNAs etc.) that reduce expression of the major UPR regulator Grp78/Bip, overexpressed by TrkAIII transfectants may target this mechanism to render TrkAIII-expressing tumour cells more sensitive to acute ER-stress-induced death. Down-regulation of Grp78/Bip expression could be achieved using the cancer drug OSU-03012, developed from Celecoxib, that supresses Grp78/Bip expression by > 90% in many tumour cell lines and significantly increases tumor cell killing [73]. Combining OSU-03012 with small molecule Trk inhibitors and agents that induce ER-stress may also slow disease progression by enhancing sensitivity to ER stress.

### Targeting anti-apoptotic Bcl-2, Bcl-xl and Mcl1 proteins

TrkAIII enhances NB cell resistance to genotoxic-, oxidative- and ER-stress by increasing the expression and mitochondrial-localisation of anti-apoptotic Bcl-2, Bcl-xL and Mcl-1 proteins [32, 58]. Therefore, agents that reduce Bcl-2, Bcl-xL and/or Mcl1 expression combined with small molecule Trk inhibitors and agents that promote mitochondrial apoptosis, would be expected to kill TrkAIII expressing tumour cells. Agents that down-regulate the expression of Bcl-2-family proteins, include: ABT-737, ABT-263 (Navitoclax) and ABT-199; WEHI-539; BXI-61 and BXI-72; Obatoclax; S1; JY-1-106; Apogossypol and derivatives [74], and agents that down-regulate Mcl-1 expression, include: Flavopiridol and SNS-032 cyclin-dependent kinase inhibitors; Sorafenib multi kinase inhibitor; WP1130 de-ubiquitinase inhibitor; antisense oligonucleotides; ABT-737 and Obatoclax BH3 mimetics, and Gossypol polyphenolic aldehyde and its derivative Sabutoclax (Bi-97C1) [75].

### Targeting the TrkAIII/SOD2 axis

TrkAIII augments the expression of mitochondrial SOD-2, enhancing resistance to mitochondrial ROS-mediated death [49]. Down regulation of SOD2 expression would be expected to compromise this important mitochondrial protection mechanism and combined with small molecule Trk inhibitors and agents that promote mitochondrial ROS production, could increase TrkAIII-expressing tumour cell-sensitivity to mitochondrial ROS-mediated death and may also target the tumour stem cell-niche, which is promoted by TrkAIII and associated with enhanced SOD2 expression [49]. MiR-509-5p has been reported to down-regulates SOD2 expression and suppresses breast cancer metastatic progression [76]. MiR-509-5p mimics, therefore, could disrupt the TrkAIII/SOD2 mitochondrial protection axis to facilitate cancer drug-induced mitochondrial ROS-mediated death. However, the different roles played by SOD2 in cancer progression, metastasis and tumour inhibition [77], must be carefully assessed when considering SOD2 as a potential target.

### Maximising sensitivity to TRAIL-induced apoptosis

The original pre-clinical promise of pro-apoptotic TRAIL/TRAIL-R signaling as a tumour-specific immunotherapy has been disappointing. Recombinant soluble TRAIL exhibits limit efficacy, short half-life and rapid clearance in vivo and first generation TRAIL-receptor agonists, designed to reduce toxicity, exhibit limited efficacy. Furthermore, many cancers including NB exhibit initial or acquired resistance to TRAIL-induced apoptosis and exogenous TRAIL may enhance the proliferation, invasive and metastatic behaviour of TRAIL-resistant tumour cells. These setbacks, however, have resulted in the development of innovative reagents and novel ways to increase half-life and overcome resistance, with major improvements in TRAIL and TRAIL receptor-agonists, delivery and ways to overcome TRAIL-resistance (reviewed in [78–80]).

**Fig. 8:**
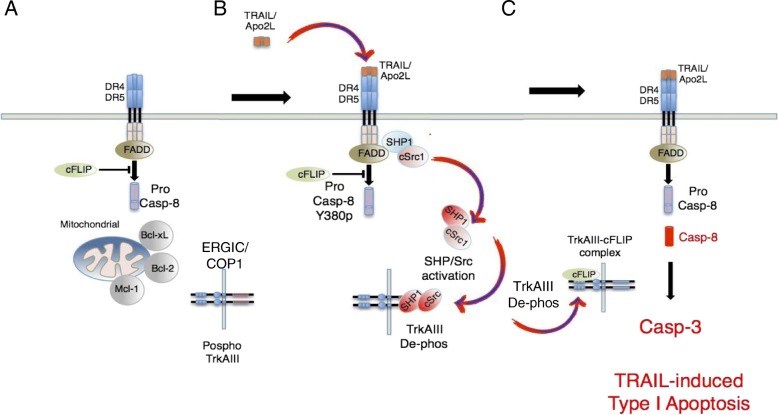
TrkAIII sensitizes NB cells to TRAIL-induced apoptosis. Schematic representation of: **a**) cFLIP and Bcl-2 protein family inhibitors of the extrinsic and intrinsic apoptosis pathways in NB cells. **b**) One-way pro-apoptotic crosstalk between the TRAIL activated TRAIL receptor pathway and TrkAIII pathways, resulting from TRAIL-induced, Shp-dependent c-Src activation, Shp-1/cSrc/TrkAIII complexing, Shp-1-dependent TrkAIII de-phosphorylation, resulting in (**c**) TrkAIII/cFLIP complexing that alters the caspase 8/cFLIP ratio at death receptors, inducing delayed apoptosis through the extrinsic pathway

TrkAIII exhibits a potential therapeutic “Achilles heel” by sensitizing transfectants to one-way TRAIL-induced pro-apoptotic crosstalk between the TRAIL receptor signaling pathway and TrkAIII, resulting in delayed apoptosis and abrogation of tumorigenic activity in vitro [58]. In cells blocked in the intrinsic apoptosis pathway by elevated Bcl-2, Bcl-xL and Mcl1 expression [32, 58], TRAIL-induces delayed apoptosis through the extrinsic pathway [58]. This initiates with TRAIL-induced SHP-mediated activation of c-Src and the formation of complexes between activated TrkAIII, SHP-1 and c-Src. This results in SHP-mediated TrkAIII de-phosphorylation and the subsequent formation of complexes between de-phosphorylated TrkAIII and cFLIP. This alters the ratio of caspase-8 to cFLIP at DR4/5 death receptors, in favour of caspase-8, resulting in delayed TRAIL-induced apoptosis. Furthermore, Mcl-1 and cFLIP play rate-limiting roles in TrkAIII transfectant-sensitivity to TRAIL-induced apoptosis, suggesting that cFLIP and/or Mcl-1 inhibitors, combined with novel TRAIL and TRAIL-receptor agonist formulations could represent an important tumour-specific immunotherapy option for TrkAIII expressing tumours (Schematized in Fig. 8). TRAIL also induces apoptosis and abrogates the in vitro tumorigenic activity of NGF-activated TrkA expressing NB cells, via a mechanism that overcomes cFLIP-mediated resistance to TRAIL-induced apoptosis in the absence of NGF, adding a novel important pro-apoptotic immunological dimension to NGF/TrkA interactions in NB cells. This effect is temporary and is subsequently inhibited by NGF-dependent, NF-κB-mediated induction of Mcl-1 expression, suggesting a potential pro-apoptotic use of painless NGF formulations and TRAIL combined with NF-κB and/or Mcl-1 inhibitors for favourable and unfavourable NBs that express fully spliced TrkA [81].

### Therapeutic potential of geldanamycin A (GA)-analogues

Hsp90 stabilises and promotes the activity of receptor tyrosine kinase (RTK) oncogenes, many of which are inhibited and/or induced to degrade by the Hsp90 inhibitor GA, prompting therapeutic evaluation and development of GA-analogues for use in adult and paediatric cancers, including NB [82]. TrkAIII also exhibits GA-sensitive interactions with Hsp90 and Grp94, critical for TrkAIII ER-export, spontaneous ERGIC/COPI-associated activation and oncogenic function but not for TrkAIII stability [50]. In contrast to GA-analogue effects on tumours driven by other RTK oncogenes, however, TrkAIII exerts a negative impact upon GA-induced NB cell eradication, increasing TrkAIII ER-accumulation, resulting in rapid attenuation UPR-associated Ire1a/Xbp-1 activation and enhanced survival, when compared to control and TrkA transfectants. In TrkA/TrkAIII transfectant co-cultures, treatment with GA results in the selection of TrkAIII transfectants, which most likely depends upon preconditioning associated with constitutive partial UPR activation (see above), pre-existing PI3K/Akt/NF-кB survival signaling and elevated Bcl-2, Bcl-xL and Mcl-1 expression, which despite GA inhibition of TrkAIII activation, enhances survival. TrkAIII PNA inhibits this effect, indicating that the therapeutic potential of GA-analogues in TrkAIII expressing tumours would require combined inhibition of TrkAIII expression.

## Conclusions

**Fig. 9:**
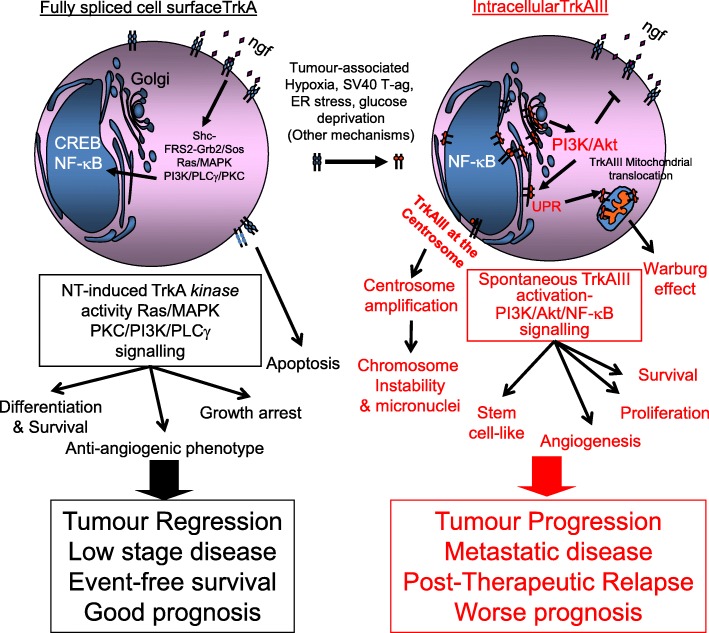
Stress-regulated alternative TrkAIII splicing promotes neural- and neural-crest-related tumour progression. Schematic representation equating the tumour suppressing TrkA effects (differentiation, growth arrest, apoptosis and inhibition of angiogenesis) to the oncogenic TrkAIII effects (proliferation, staminality, stress resistance, survival, angiogenesis, genetic instability and the Warburg effect) resulting from D4 domain-deletion, receptor re- intracellular localisation, PIP3K/Akt/NF-κB, centrosome amplification and ER-stress induced translocation to and activation within the mitochondria

**Fig. 10:**
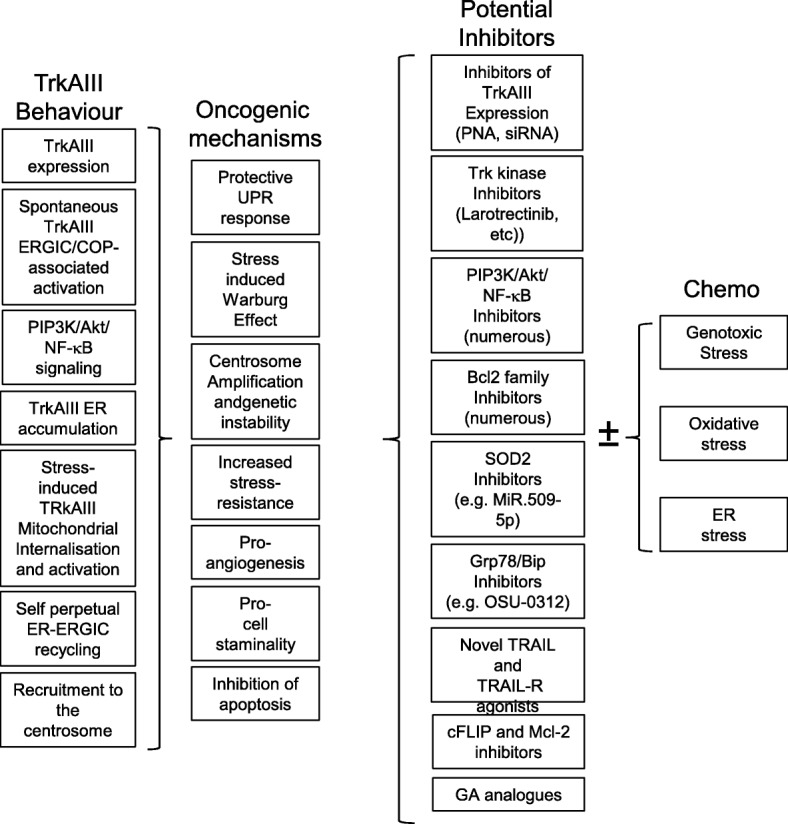
Potential TrkAIII-targeted and alternative therapeutic approaches to TrkAIII expressing cancers. Representation of TrkAIII behaviour, oncogenic mechanisms and potential therapeutic inhibitors of TrkAIII activity, expression and down stream signaling, which either alone or in combination with genotoxic-, oxidative and/or ER stress-inducing cancer drugs may be of therapeutic benefit in TrkAIII expressing cancers

The profound, long-term therapeutic responses reported for TrkA fusion oncogene-driven cancers to Trk inhibitory therapy, highlights the need to better identify cancers driven by TrkA oncogenes, including TrkA oncogenes activated by aberrant alternative *TrkA* splicing, such as TrkAIII. The capacity of alternative splicing to unlock TrkA oncogenic potential, characterises sequence encoded within exons 6–7 in preventing oncogenic activation, the deletion of which causes receptor re-localization and spontaneous activation within altered intracellular substrate contexts, resulting in a plethora of “addiction” and “non-addiction” oncogenic mechanisms that impact upon a majority of the Hallmarks of Cancer (Schematized in Figs. 7 and 9). We propose, therefore, that efforts should be doubled to identify cancers potentially driven by aberrant alternative TrkAIII splicing in order to extend potential therapeutic options to include small molecule Trk tyrosine kinase inhibitors, inhibitors of PI3K/Akt/NF-кB signaling, inhibitors of Bcl-2 protein family and/or SOD2 expression, reversal of alternative TrkAIII splicing or novel TRAIL or TRAIL-receptor agonist formulations. These approaches combined with existing genotoxic-, oxidative- and/or ER-stress inducing cancer drugs could result in important long-term therapeutic responses (Summarised in Fig. 10). Finally, alternative oncogenic *TrkA* splicing may not be limited to TrkAIII [17, 22], suggesting that characterisation of all oncogenic alternative *TrkA* splice variants may be required to fully exploit the therapeutic potential of Trk tyrosine kinase inhibitors and/or inhibitors of post receptor signaling.

## Abbreviations

2DG: 2-deoxy-glucose
Alk: Anaplastic lymphoma kinase
ATF6: Activating transcription factor 6
Bcl2: B cell lymphoma 2
Bcl-xL: B cell lymphoma extra large
cFLIP: Cellular FLICE (FADD-like IL1β-converting enzyme)-inhibitory protein
CHOP: C/EBP homologous protein
COP: Coatomer protein
DTT: Dithiothreitol
EGFR: Epithelial growth factor receptor
EGFRvIII: Epithelial growth factor receptor variant III
ER: Endoplasmic reticulum
ERGIC: Endoplasmic reticulum Golgi-intermediate compartment
GA: Geldanamycin A
GN: Golgi network
Grp78/BiP: Glucose related protein 78/binding immunoglobulin protein
Grp94: Glucose-related protein 94
HSP90: Heat shock protein 90
IMM: Inner mitochondrial membrane
Ire1a: Inositol requiring enzyme 1
IкB: Inhibitor kappa binding
MAM: Mitochondrial-associated ER membranes
MAPK: Mitogen activated protein kinase
Mcl-1: Myeloid leukemia cell differentiation protein
MiR: Micro RNA
MMP: Matrix metalloproteinase
MT: Microtubules
MTOC: Microtubule organising centre
NB: Neuroblastoma
NF-кB: Nuclear factor kappa-binding
NGF: Nerve growth factor
NT-3: Neurotrophin-3
Omi/HTRA2: High temperature requirement factor 2
OMM: Outer mitochondrial membrane
PDHK1: Pyruvate dehydrogenase kinase 1
PDTC: Ammonium pyrrolidinedithiocarbamate
PERK: Pancreatic endoplasmic reticulum kinase
PI3K: Phosphoinositol-3-kinase
Plk4: Polo kinase 4
PNA: Peptide nucleic acid
siRNA: Small inhibitory ribonucleic acid
SOD2: Superoxide dismutase 2
SV40: Simian virus 40
TIMP: Tissue inhibitor of metalloproteinases
TRAIL: Tumor necrosis factor-related apoptosis-inducing ligand
TRAIL-R: Tumor necrosis factor-related apoptosis-inducing ligand receptor
TrkA: Tropomyosin related kinase A
Tsp1: Thrombospondin 1
UPR: Unfolded protein response
VEGF: Vascular endothelial cell growth factor
Xbp1: Xbox binding protein 1
